# Action Mechanisms of Metformin Combined with Exenatide and Metformin Only in the Treatment of PCOS in Obese Patients

**DOI:** 10.1155/2023/4288004

**Published:** 2023-12-14

**Authors:** Jingwen Gan, Jie Chen, Rui-lin Ma, Yan Deng, Xue-song Ding, Shi-yang Zhu, Ai-jun Sun

**Affiliations:** ^1^National Clinical Research Center for Obstetric & Gynecologic Diseases, Department of Obstetrics and Gynecology, Peking Union Medical College Hospital, Chinese Academy of Medical Sciences & Peking Union Medical College, No. 1 Shuaifuyuan, Dongcheng District, Beijing 100730, China; ^2^Department of Ultrasound, Qilu Hospital of Shandong University, Jinan, Shandong 250012, China

## Abstract

**Background:**

Polycystic ovary syndrome (PCOS) is the most common endocrine disease in women of reproductive age, whose clinical characteristics are hyperandrogenism (HA), ovulatory dysfunction, and polycystic ovary, often accompanied by insulin resistance (IR) and metabolic abnormalities. Glucagon-like peptide (GLP)-1 receptor agonists (GLP-1Ra), such as exenatide, can bind to specific receptors on tissues such as the ovaries to improve the clinical phenotype of PCOS, while insulin-sensitizing agents, such as metformin, can also benefit to metabolic abnormalities in PCOS. Liquid chromatography-mass spectrometry (LC/MS) metabolomics revealed differences between the mechanisms of exenatide and metformin treatment of PCOS to some extent.

**Methods:**

In this study, 50 obese subjects with PCOS were randomly divided into the exenatide combined with metformin group (COM group, *n* = 28) and the metformin group (MF group, *n* = 22) for 12-week treatment. Before and after, serum samples were subjected to LC/MS analysis.

**Results:**

After treatment, there were 153 named differential metabolites in the COM group and 99 in the MF group. Most phosphatidylcholines (PC) and deoxycholic acid 3-glucuronide (DA3G) were significantly upregulated, while most glycerophosphoethanolamine (PE-NMe2), glycerophosphocholine (GPC), and threonine were downregulated in both groups. Only the decrease of neuromedin B, glutamate, and glutamyl groups and the increase of chenodeoxycholic acid sulfate docosadienoate (22: 2n6), and prostaglandin E2 have been observed in the COM group. In addition, salicylic acid and spisulosine increased and decanoylcarnitine decreased in the MF group. Both groups were enriched in glycerophospholipid, choline, and sphingolipid metabolism, while the COM group was especially superior in the glutamine and glutamate, bile secretion, and amino acid metabolism.

**Conclusion:**

Compared with metformin alone in the treatment of PCOS, the differential metabolites of the exenatide combined with metformin group are more extensive. The COM group may act on the hypothalamic-pituitary-gonadal axis (HPO) and its bypass, regulate multiple metabolism pathways such as phospholipids, amino acids, fatty acids, carnitine, bile acids, and glucose directly or indirectly in obese PCOS patients.

## 1. Introduction

Polycystic ovary syndrome (PCOS) is a heterogeneous disease with clinical manifestations of hyperandrogenic signs, ovulation disorders, and oligomenorrhea. Meanwhile, 50%–70% of PCOS patients with reproductive and endocrine metabolic disorders, such as insulin resistance (IR) [1], and obese PCOS patients are more prone to infertility, diabetes, and cardiovascular disease [2]. In addition to adjusting lifestyle, metformin, a classic hypoglycemic drug, is commonly used clinically in the treatment of PCOS patients with impaired glucose tolerance or metabolic syndrome who are ineffective in simple lifestyle adjustment [3]. It can inhibit hepatic gluconeogenesis, promote peripheral glucose uptake, and reduce fatty acid oxidation, thereby improving liver sensitivity to insulin [4]. Exenatide is a glucagon-like peptide (GLP)-1 receptor agonist (GLP-1Ra) analog, which can inhibit appetite and gastrointestinal peristalsis [5]. It can also help in weight loss, improve IR, and reduce the risk of cardiovascular disease [6]. At present, many studies have confirmed the efficacy and safety of exenatide in type 2 diabetes mellitus (T2DM) [7], and recent studies have recommended exenatide alone or in combination with metformin are good options for PCOS women with obesity, glucose intolerance or high risks of CVD [8]. However, there are few reports about the metabolic differences between metformin and exenatide.

Metabolomics is a helpful tool to identify small metabolite molecules. Compared to genomics and proteomics, metabolomics is directly influenced by factors such as genetics and environment, which means it can clearly reveal the exact metabolic changes that occur in the body under a certain disease state (such as physiological or pathological stimuli, before and after administration, etc.). By studying those differences, the biomarkers of the body in a specific state are clarified, which provides a new direction for clinical diagnosis and treatment [9].

This study intends to summarize the value and pharmacodynamics of glucagon-like peptide receptor agonists and insulin sensitizers by analyzing the metabolite changes after exenatide combined with metformin or single metformin treatment in PCOS patients who are obese.

## 2. Subjects and Methods

This study was conducted in accordance with the Declaration of Helsinki and was approved by the Ethics Committee of Peking Union Medical College, Chinese Academy of Medical Sciences, and Peking Union Medical College (No. HS-2032). Informed consent forms were signed by all subjects before the start of the study. Obese PCOS patients were selected from Peking Union Medical College Hospital from July 2019 to January 2021. PCOS was diagnosed based on the presence of two out of three criteria (oligomenorrhoea, clinical or biochemical hyperandrogenism, and polycystic ovaries on ultrasound after exclusion of other endocrine causes of hyperandrogenism according to the Rotterdam criteria) [10]. They were aged 18–40, overweight or obese (body mass index ≥ 25 kg/m^2^), took no regular medications known effects on reproductive or metabolic function, have no other endocrine diseases, no other malignancies, no multiple endocrine neoplasia type 2 (MEN2) or personal or family history of medullary thyroid carcinoma (MTC), no history of thromboembolic disease or thrombosis, no serious or unstable physical diseases, no drug allergy, not in other clinical trials, not breastfeeding or pregnant, and nonheavy smokers or alcoholics. All the subjects had anthropometric measurements and fasting blood sampling.

A total of 70 patients were recruited to receive randomized treatment, and 50 completed the trial due to the impact of the COVID epidemic. Therein, patients in the metformin group (MF group, *n* = 22) received metformin 500 mg alone, three times a day; others in the exenatide combined with the metformin group (COM group, *n* = 28) received a subcutaneous injection of exenatide 2 mg once a week in addition to regular metformin treatment. The waist circumference and body weight were measured before and after 12-week treatment, and the body mass index (BMI) was calculated in both groups., A standard 75 g oral glucose tolerance test (OGTT) was performed in the morning after overnight fasting for 12 hours, respectively. At 0, 60, and 120 min, blood glucose and insulin levels were measured. Fasting blood samples taken on the 2nd or 3rd of the patients' menstrual cycle were also used to measure hemoglobin A1C (HbA1c), sex hormones (total testosterone, TT, and dehydroepiandrosterone sulfate, DHAS), blood lipids, complete blood count, C-reactive protein, and markers of liver and kidney function. During the period, patients recorded adverse reaction events in the patient diary.

### 2.1. Analysis of Untargeted Metabolomics

At baseline and after 12 weeks of treatment, 2 tubes of venous blood were collected from eligible patients (*n* = 50) with a 4 ml vacuum blood collection tube, for a total of 8 ml. Samples were placed in a refrigerator at 4°C for 1 hour, centrifuged at 3000 rpm for 5 minutes, and the supernatant was dispensed into 1.5 ml EP tubes and immediately stored in a −80°C refrigerator until all specimens were collected. After serum samples were centrifuged at 13000 g at 4°C for 15 min, the supernatant was carefully transferred to sample vials for LC-MS/MS analysis. A pooled quality control sample (QC) was fulfilled by mixing equal volumes of all samples in order to monitor the stability of the analysis.

The metabolomic analysis of the blood samples was performed using a liquid chromatography-mass spectrometer *Q* Exactive (Thermofisher), including positive and negative ion detection modes.

(1) Liquid chromatography conditions: the column was BEH Amide (2.1 mm × 100 mm, waters, USA), the column temperature was set to 35°C, and the flow rate was 300 *µ*L/min. (2) Mass spectrometry conditions: a heated electrospray ion source (HESI) was used, the temperature of the ion transfer tube was 320°C, the temperature of the auxiliary gas was 300°C, and the flow rates of the sheath gas and the auxiliary gas were 35 and 10 (arb), respectively. Data acquisition was performed in a lazy scanning mode (Full MS/dd-MS2). Data acquisition was divided into positive ion mode (3500 V) and negative ion (−2800 V).

Collected data were secondarily matched to local databases (established based on chemical standards or biological samples) by Tracerfinder 3.2 (Thermo Fisher, CA). Metabolites after matching and identification will have a secondary score (library score, LS). Only metabolites with an LS score higher than 30 are considered credible. The higher the score, the more credible the identified metabolites.

### 2.2. Data Analysis

Metabolite data were normalized prior to statistical analysis, with peak areas normalized to the mean of the total sample area. Metabolite data were imported into SIMCA (Umetrics, Sweden) software for analysis. Principal component analysis (PCA) and supervised orthogonal partial least squares discriminant analysis (OPLS-DA), cross-validation analysis of variance (CV-ANOVA), R2Y, and the *Q*^2^ value assessed the quality of the OPLS-DA model and generated the corresponding VIP metabolite list by the OPLS-DA model. *t*-test results and FDR correction were performed with Metaboanalyst 3.0 (https://www.metaboanalyst.ca/), and variables with VIP >1 and FDR <0.05 were considered as the most likely differential metabolites. Pathway analysis was performed through the human metabolome database (https://www.HMDB.ca/), the small molecule pathway database (SMPDB) and the Metaboanalyst 3.0 (https://www.metaboanalyst.ca/) metabolomics analysis platform.

Statistical analysis was performed by the SPSS version 25.0. Quantitative demographic and clinical data with normal distribution in the two groups were expressed as the mean ± standard deviation and analyzed by Student's *t*-test. Paired sample *t*-test for normally distributed data within the group and independent-samples *t*-test when comparing between groups. The quantitative sequencing data with a non-normal distribution were analyzed by the Wilcoxon rank sum test. For all analyses, a two-tailed *P* ≤ 0.05 was considered to indicate statistical significance.

## 3. Results

### 3.1. Comparison of Clinical Characteristics and Biochemical Data

Clinical characteristics and biochemical data of subjects at the baseline and after intervention are shown in Table 1. Although BMI and androgen in both groups were significantly decreased after treatment, there is no difference between the two groups. In terms of glucose metabolism, the 2h-OGTT and HbA1c of the COM group reduced significantly, while the level of the MF group did not drop compared to baseline, and the COM group had a better result in alleviating the level of FINS than the MF group. We observed changes in some indicators of lipid metabolism in each group; TG, TC, and LDL levels did not significantly change from baseline in any group. HDL and ApoA1 have markedly improved from baseline in both groups, and only the MF group had lower lipoprotein B (ApoB) after treatment. In our study, TT was significantly decreased from baseline in each treatment group and DHAS levels did not change significantly.

### 3.2. Metabolic Profiling Analysis

#### 3.2.1. General Characteristics of Serum Metabolic Profiling Changes in the Two Groups

**Figure 1:**
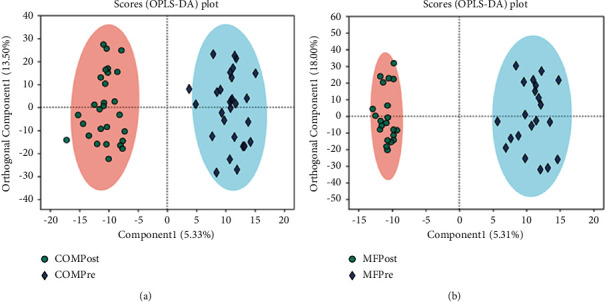
General characteristics of serum metabolites in two groups. OPLS-DA score map: the first prediction of Comp1 is mainly the decomposition degree, and the first orthogonality of orthogonal Comp1 is the decomposition degree. Scores map of the OPLS-DA model before and after treatment to obese PCOS women in the COM group (a) (*R*^2^*X* = 0.341, *R*^2^*Y* = 0.95, *Q*^2^ = 0.458) and in the MF group (b) (*R*^2^*X* = 0.398, *R*^2^*Y* = 0.973, *Q*^2^ = 0.668).

The OPLS-DA model was also used to analyze the changes in the distribution of serum metabolites in the two groups before and after treatment. Serum metabolites in the COM and MF group separated significantly (Figure 1).

#### 3.2.2. Serum Metabolite Changes in Obese PCOS Patients after Treatment

**Figure 2:**
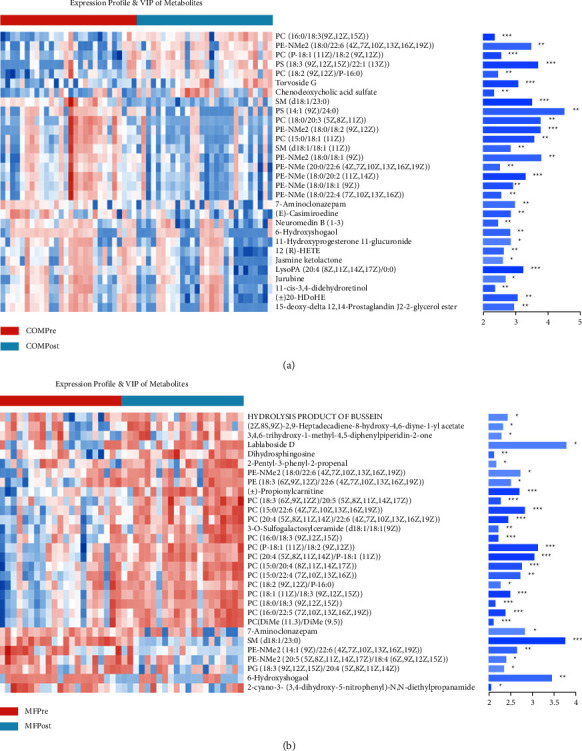
Heatmaps of the top 30 differential metabolites in the COM and MF groups before and after treatment. Heatmap: the color indicates the relative expression level of the metabolite in this group of samples. The corresponding relationship between the color gradient and the numerical value is shown in the gradient color block. The right side is the metabolite VIP bar chart. The length of the bar represents the contribution value of the metabolite to the difference between the two groups (VIP > 1), the larger the value, the greater the difference between the two groups. The color of the bar indicates the significance of the difference between the two groups of metabolites, namely, the *P* value. The smaller the *P* value is, the darker the color is. ^*∗*^*P* < 0.05, ^*∗∗*^*P* < 0.01, ^*∗∗∗*^*P* < 0.001. (a) Heatmap of the top 30 VIP metabolites that were differentially expressed in the COM group. (b) Heatmap of the top 30 VIP metabolites that were differentially expressed in the MF group.

A total of 2055 differential metabolites were detected, and 153 named differential metabolites were successfully quantified in the COM group (*p* < 0.05, variable importance for the projection (VIP) > 1, Figure 2(a)), including 86 upregulated and 53 downregulated metabolites after treatment. We observed that phosphatidylcholines (PC), chenodeoxycholic acid sulfate, deoxycholic acid 3-glucuronide (DA3G), docosadienoate (22: 2n6), and prostaglandin E2 were increased after treatment, while phosphatidylethanolamine (PE-NMe2), lysophosphatidic acid (LPA), neuromedin B, glutamate and Glutamyl groups, and threonine and spisulosine, were decreased after treatment.

While the MF group had been identified, a total of 1655 differential metabolites (*p* < 0.05, VIP > 1, Figure 2(b)) and 99 metabolites were named, which including 71 upregulated and 28 downregulated metabolites after treatment. We observed that PC, DA3G, salicylic acid, and spisulosine were upregulated after treatment; phosphatidylserine (PS), sphingomyelins (SM), glycerophosphocholine (GPC), threonine, and decanoylcarnitine were downregulated after the treatment. PE-NMe2s had different trends in the MF group (Figure 2(b)).

**Figure 3:**
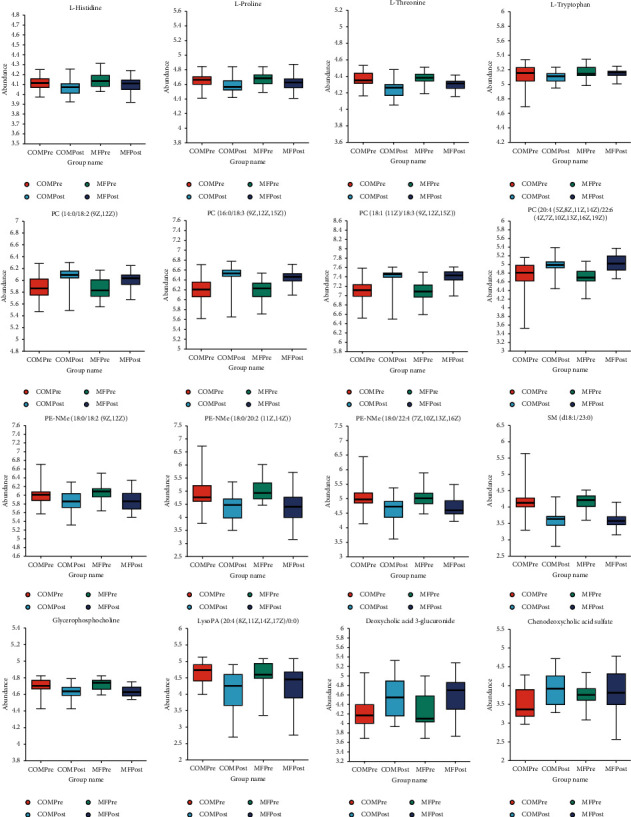
Box diagram of differential metabolites with the same change trend between groups before and after treatment.

We also compared the change in serum metabolites in obese PCOS patients between groups. We had observed certain metabolites, including PCs, PE-NMe2s, GPC, DA3G, chenodeoxycholic acid sulfate, and certain amino acids, had similar improving trend in both groups (Figure 3).

### 3.3. Enrichment Analysis on Differential Metabolites and Differential KEGG Pathways

**Figure 4:**
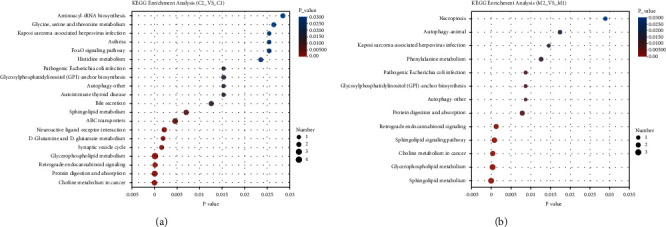
KEGG pathway analysis of the differentially expressed metabolites. The abscissa is the *P* value of enrichment significance. The smaller the *P* value, the more significant it is in statistics. The ordinate is the KEGG pathway. The size of the bubbles in the figure represents the number of metabolites in the pathway that are enriched in the metabolic set. (a) Bubble chart in the COM group before and after treatment. (b) Bubble chart in the MF group before and after treatment.

KEGG pathway enrichment analysis of the differential metabolites in the COM and MF groups showed that a total of 31 metabolic pathways were observed to be enriched in the COM group and only 12 in the MF group (Figure 4). Both groups were enriched in glycerophospholipid metabolism, choline metabolism, sphingolipid metabolism, protein digestion, and absorption. While metabolite changes in the COM group were also associated with glutamine and glutamate metabolism, bile secretion, and amino acid metabolism. Only phenylalanine metabolism showed in the MF group.

## 4. Discussion

Exenatide could significantly reduce body weight, insulin, testosterone, and interleukin 6 (IL-6) levels in the rat model and also improve changes in ovarian morphology, whose effect was similar to that of metformin [12]. The decrease of the body weight and central adiposity by exenatide may further explain improvements in IR, inflammatory markers, and menstrual cycles [13]. Some clinical studies comparing GLP-1 RA with metformin showed that the former has advantages in improving body weight, HOMA-IR, menstrual frequency, and spontaneous pregnancy rate [14]. The investigators further suggested that exenatide combined with metformin appeared to have a greater (additive) effect than exenatide or metformin in terms of menstrual cycle frequency, hormonal and metabolic disturbances [8], and remission rates for prediabetes [15].

However, most of the current articles mainly compare the efficacy of exenatide and metformin in the treatment of PCOS patients from the perspective of clinical biochemical data. Tang et al. [16] first analyzed the metabolites after exenatide therapy in OB/OW PCOS patients and found that lipid and amino acid metabolic abnormalities and steroid and nucleotides synthesis might be improved after exenatide treatment. In our study, we showed that exenatide combined with metformin have a better effect on remising OB/OW PCOS patients by improving multiple metabolisms.

Phosphatidylcholine (PC), glycerophospholipids (GPC), and phosphatidylethanolamine (PE) in PCOS patients are lower than those in healthy people [17–19]. In our study, the COM group and MF group both significantly increased PC and deoxycholic acid 3-glucuronide (DA3G), while most glycerophosphoethanolamine (PE-NMe2), glycerophosphocholine (GPC), and threonine were downregulated after treatment. PC can promote cell proliferation and programmed cell death and improve insulin sensitivity [20]. Blood phosphatidylcholine can be used as a chemical attractant to recruit immune cells such as macrophages and dendritic cells, thereby improving immune function [21]. These results were consistent with the conclusion of Yao et al. [22], transplanting the brown adipose tissue in PCOS model mice. GPC/PC was significantly decreased in OB/OW PCOS patients, which was contrary to the conclusion of the previous study [18].

Previous studies have proved that the increase of the branched-chain amino acid (BCAA) levels affected women with PCOS due to the protein phosphatase Mg^2+^/Mn^2+^-dependent 1K (PPM1K) deficiency. In nonobese PCOS patients, arginine, lysine, ornithine, proline, glutamate, acetone, citrate, and histidine decreased significantly [23], and in those with obesity, specially, branched-chain amino acids (e.g., leucine) and aromatic amino acids (e.g., phenylalanine) were significantly lower than in healthy people [16, 18]. However, we found that both groups had lower levels of many amino acids like histidine, proline, threonine, and tryptophan, especially in the COM group, which suggested that exenatide combined with metformin played an important part in the amino acid metabolism. Neurokinin B (NKB) is a peptide neurotransmitter regulated by KNDy neurons in the hypothalamus. Studies suggested that the abnormal activation of KNDy neurons could regulate the autocrine and/or paracrine of kisspeptin, NKB, and dynorphin (Dyn), which might increase the GnRH pulse frequency and resultantly LH secretion, involving the pathology of PCOS [24]. At present, only Gorkem et al. [25] reported that kisspeptin in PCOS patients was significantly increased, and none have reported the changes of NKB in PCOS patients. We found out that NKB levels decreased after treatment only in the COM group, suggesting that exenatide may downregulate KNDy neuronal activity, namely, hypothalamic-pituitary-gonadal axis (HPO) or its passby, thereby improving the PCOS phenotype.

Besides, PCOS patients had higher *n* − 6 series polyunsaturated fatty acids (PUFAs) than healthy women, while *n* − 3 series PUFAs were lower. Our results showed that docosatrienoic acid, a kind of *n* − 3 series PUFAs, and prostaglandin E2 (PGE2) elevated after treatment in the COM group only. *n* − 3 series PUFAs have a protective effect on insulin sensitivity [26, 27], inhibit the synthesis of androgens in PCOS patients, and promote the synthesis of estradiol and progesterone [28, 29]. PGE2 is a lipid-derived substance that has been shown to regulate the function of many cell types, including dendritic cells, macrophages, and T and B lymphocytes, leading to proinflammatory and anti-inflammatory effects [30]. What's more, we noticed that the (E)-Casimiroedine level, belonging to glycosylamines, was only reduced in the COM group. Glycosylamines are valuable sugar derivatives that have attracted much attention as synthetic intermediates en route to iminosugar-C-glycosyl compounds, which work as inhibitors of a wide variety of glycosidases and glycosyltransferases [31], leading to glucose metabolism disorders.

In addition, our study showed that chenodeoxycholic acid sulfate (CDCA) was increased in the COM group after treatment. Jia et al. [32] first found that glycocholic acid, which can dissolve fat, was reduced in PCOS patients; therefore, these patients have problems with fat absorption. CDCA is the most potent bile acid (BA) for activating the farnesoid X receptor (FXR) [33]. FXR not only effectively regulates bile acid synthesis and secretion and lipid and glucose metabolism in the liver and intestine [34] but also counteracts the proinflammatory and proatherosclerotic responses of cardiovascular disease, modulates liver inflammation and regeneration, modulates the degree of inflammatory response, and inhibits barrier function and intestinal bacterial translocation [35]. When CDCA increases, PCOS phenotypes can be alleviated due to the activation of FXR. Therefore, exenatide combined with metformin could directly and indirectly improve glucose and lipid metabolism more effectively compared to the MF group.

## 5. Conclusion

Exenatide combined with metformin had more differential metabolites compared to metformin alone, acting positively and effectively on the HPO and its bypass, improving amino acid, glucose, and lipid metabolism directly or indirectly, thereby inhibiting androgen synthesis, improving insulin resistance, and other metabolic syndromes in obese PCOS patients.

## Figures and Tables

**Table 1:** Clinical characteristics and biochemical data between the COM and MF group.

	COM group			MF group		
	Before	After	Δ (before—after)	Before	After	Δ (before—after)
Age (years)	29.96 ± 5.39	—	—	28.45 ± 4.74	—	—
BMI (kg/m^2^)	32.30 ± 3.87	31.21 ± 3.86^##^	1.09 ± 1.09	29.90 ± 2.66	29.37 ± 2.85^##^	0.53 ± 0.80
WHR	0.91 ± 0.07	0.91 ± 0.05	0.0066 ± 0.056	0.91 ± 0.07	0.90 ± 0.07	0.0057 ± 0.036
LH (IU/L)	6.61 ± 3.52	10.54 ± 12.94	−3.93 ± 14.96	8.66 ± 5.76	10.85 ± 6.65	−2.18 ± 6.93
FSH (IU/L)	5.73 ± 2.33	55.60 ± 37.72	−1.98 ± 3.80	5.69 ± 1.97	7.37 ± 1.57	−1.67 ± 2.86
E_2_ (pg/ml)	7.72 ± 2.83	81.20 ± 51.07	25.60 ± 76.26	59.44 ± 34.96	53.78 ± 17.62	25.66 ± 34.12
TT (ng/ml)	0.72 ± 0.31	0.59 ± 0.21^##^	0.13 ± 0.21	0.71 ± 0.22	0.55 ± 0.17^#^	0.15 ± 0.20
DHAS (*μ*g/dl)	253.39 ± 74.15	252.69 ± 88.43	0.70 ± 50.15	267.45 ± 115.39	260.30 ± 110.99	7.15 ± 47.09
FBG (mmol/L)	5.16 ± 0.59	5.02 ± 0.45	0.15 ± 0.42	5.25 ± 0.42	5.28 ± 0.45	−0.26 ± 0.30
OGTT-2h BG (mmol/L)	7.90 ± 1.44	7.40 ± 1.87	0.49 ± 2.37^*∗*^(*p*=0.008)	7.24 ± 1.66	8.48 ± 1.56^##^	−1.24 ± 1.46
FINS (*μ*IU/ml)	26.29 ± 14.38	22.69 ± 9.37	3.60 ± 12.18^*∗*^(*p*=0.034)	20.04 ± 7.15	23.53 ± 10.28	−3.47 ± 7.71
OGTT‐2h INS (*μ*IU/ml)	198.99 ± 85.58^*∗*^	156.46 ± 92.59	42.53 ± 105.85	149.43 ± 67.21	131.49 ± 62.20	17.93 ± 79.25
HbA1c	5.46 ± 0.47	5.34 ± 0.42^#^	0.12 ± 0.24	5.34 ± 0.32	5.31 ± 0.21	0.03 ± 0.23
HOMA-IR	6.17 ± 3.67	5.01 ± 1.94	1.16 ± 3.29^*∗*^ (*p*=0.017)	4.53 ± 1.88	5.38 ± 2.56	−0.85 ± 1.84
TC (mmol/L)	4.99 ± 0.85	5.29 ± 1.06	−0.30 ± 0.87	5.19 ± 0.90	5.32 ± 0.96	−0.53 ± 0.79
TG (mmol/L)	2.09 ± 1.64	2.48 ± 0.87	−0.39 ± 1.53	2.26 ± 1.78	2.89 ± 1.60	−0.62 ± 1.91
HDL-c (mmol/L)	1.23 ± 0.32	1.43 ± 0.32^##^	−0.20 ± 0.26	1.17 ± 0.20	1.46 ± 0.32^##^	−0.29 ± 0.20
LDL-c (mmol/L)	3.08 ± 0.65	3.07 ± 0.85	0.007 ± 0.813	3.36 ± 0.69	3.33 ± 0.86	0.02 ± 0.76
ApoA1 (g/L)	1.38 ± 0.34	1.65 ± 0.35^##^	−0.28 ± 0.34	1.28 ± 0.12	1.71 ± 0.36^##^	−0.43 ± 0.31
ApoB (g/L)	1.00 ± 0.17	0.99 ± 0.18^*∗*^	0.006 ± 0.176	1.03 ± 0.20	1.11 ± 0.20^#^	−0.082 ± 0.18
LP(a) (mg/dL)	165.86 ± 203.27	146.68 ± 203.30^*∗*^	19.18 ± 56.22	97.00 ± 90.44	53.00 ± 52.87^##^	44.00 ± 51.81
FFA (*μ*mol/L)	605.59 ± 219.38	647.59 ± 220.25	−42.00 ± 215.88	694.60 ± 187.18	678.25 ± 219.07	16.35 ± 295.87
hsCRP (mg/L)	5.01 ± 4.46	4.73 ± 4.27	0.28 ± 4.17	3.69 ± 2.56	3.90 ± 2.90	−0.21 ± 3.63
WBC (^*∗*^10^/L)	7.00 ± 1.19	7.24 ± 1.85	−0.24 ± 1.37^*∗*^ (*p*=0.019)	7, 46 ± 1.17	6.81 ± 1.03^#^	0.65 ± 1.01
ALT (U/L)	37.92 ± 31.01	29.32 ± 20.26	8.60 ± 19.35	32.57 ± 17.28	37.05 ± 46.09	−4.48 ± 38.16
Cr (*μ*mol/L)	60.96 ± 9.51	59.13 ± 8.17	1.83 ± 5.68	60.33 ± 6.06	56.71 ± 7.63^#^	3.62 ± 5.2

BMI, body mass index; WHR, waist-hip ratio; FBG, fasting blood glucose; TT, total testosterone; DHAS, dehydroepiandrosterone sulfate; OGTT, oral glucose tolerance test; HbA1c, hemoglobin A1C; Fins, fasting serum insulin; OGTT-2h INS, 2-hour insulin in OGTT. HOMA-IR, homeostasis model assessment-insulin resistant; TC, total Cholesterol; TG, triglycerides; HDL-c: high-density lipoprotein cholesterol; LDL-c, low-density lipoprotein cholesterol; ApoA1, lipoprotein A1; ApoB, lipoprotein B; LP(a), lipoprotein-a; FFA, free fatty acid; hsCRP, high-sensitivity C-reactive protein; DC, white blood cell count; ALT, alanine aminotransferase; Cr, creatinine. Homeostasis model assessment-Insulin resistant (HOMA-IR) = fasting serum insulin (mIU/L) × fasting blood glucose (FBG, mmol/L)/22.5 [11]. ^*∗*^Significant difference between the COM group and MF group (^*∗*^*P* < 0.05, ^*∗∗*^*P* < 0.01). ^#^Significant increase or decrease after treatment in the COM group or MF group (^#^*P* < 0.05, ^##^*P* < 0.01).

## Data Availability

All the data have been added to this manuscript.

## Abbreviations

PCOS: Polycystic ovary syndrome
IR: Insulin resistance
HA: Hyperandrogenism
GLP: Glucagon-like peptide
LC/MS: Liquid chromatography-mass spectrometry
GLP-1Ra: Glucagon-like peptide-1 receptor agonist
BMI: Body mass index
WHR: Waist-hip ratio
FBG: Fasting blood glucose
DHAS: Dehydroepiandrosterone sulfate
TT: Total testosterone
HbA1c: Hemoglobin A1C
FSH: Follicle-stimulating hormone
LH: Luteinizing hormone
TC: Total cholesterol
TG: Triglyceride
TT: Total testosterone
DHAS: Dehydroepiandrosterone sulfate
OGTT: Oral glucose tolerance test
HOMA-IR: Homeostasis model assessment-insulin resistant
HDL-c: High density lipoprotein cholesterol
LDL-c: Low density lipoprotein cholesterol
ApoA1: Lipoprotein A1
ApoB: Lipoprotein B
LP(a): Lipoprotein-a
FFA: Free fatty acid
hsCRP: High-sensitivity C-reactive protein
DC: White blood cell count
ALT: Alanine aminotransferase
Cr: Creatinine
PCA: Principle component analysis
OPLS-DA: Orthogonal partial least squares discriminate analysis
VIP: Variable importance for the projection
PC: Phosphatidylcholine
PS: Phosphatidylserine
PE-NMe2: Phosphatidylethanolamine
KGN: Granulosa-like tumor cell line
GPC: Glycerophosphocholine
NKB: Neurokinin B
FFA: Free fatty acid
PUFA: Polyunsaturated fatty acid
T2DM: Type 2 diabetes mellitus
CDCA: Chenodeoxycholic acid sulfate
FXR: Farnesoid X receptor.

## References

[1] González F. (2012). Inflammation in Polycystic Ovary Syndrome: underpinning of insulin resistance and ovarian dysfunction. *Steroids*.

[2] Cena H., Chiovato L., Nappi R. E. (2020). Obesity, polycystic ovary syndrome, and infertility: a new avenue for GLP-1 receptor agonists. *Journal of Clinical Endocrinology and Metabolism*.

[3] Duan X., Zhou M., Zhou G., Zhu Q., Li W. (2021). Effect of metformin on adiponectin in PCOS: a meta-analysis and a systematic review. *European Journal of Obstetrics and Gynecology and Reproductive Biology*.

[4] Escobar-Morreale H. (2018). Polycystic ovary syndrome: definition, aetiology, diagnosis and treatment. *Nature Reviews Endocrinology*.

[5] Meier J. (2012). GLP-1 receptor agonists for individualized treatment of type 2 diabetes mellitus. *Nature Reviews Endocrinology*.

[6] Garg S. (2010). The role of basal insulin and glucagon-like peptide-1 agonists in the therapeutic management of type 2 diabetes--a comprehensive review. *Diabetes Technology and Therapeutics*.

[7] Nauck M. A., Quast D. R., Wefers J., Meier J. J. (2021). GLP-1 receptor agonists in the treatment of type 2 diabetes-state-of-the-art. *Molecular Metabolism*.

[8] Siamashvili M., Davis S. N. (2021). Update on the effects of GLP-1 receptor agonists for the treatment of polycystic ovary syndrome. *Expert Review of Clinical Pharmacology*.

[9] Kennedy A. D., Wittmann B. M., Evans A. M. (2018). Metabolomics in the clinic: a review of the shared and unique features of untargeted metabolomics for clinical research and clinical testing. *Journal of Mass Spectrometry*.

[10] Rotterdam Eshre (2004). Revised 2003 consensus on diagnostic criteria and long-term health risks related to polycystic ovary syndrome. *Fertility and Sterility*.

[11] Matthews D. R., Hosker J. P., Rudenski A. S., Naylor B. A., Treacher D. F., Turner R. C. (1985). Homeostasis model assessment: insulin resistance and beta-cell function from fasting plasma glucose and insulin concentrations in man. *Diabetologia*.

[12] Sun L., Ji C., Jin L. (2016). Effects of exenatide on metabolie changes, sexual hormones, inflammatory cytokines, adipokines, and weight change in a DHEA-treated rat model. *Reproductive Sciences*.

[13] Liu X., Zhang Y., Zheng S. Y. (2017). Efficacy of exenatide on weight loss, metabolic parameters and pregnancy in overweight/obese polycystic ovary syndrome. *Clinical Endocrinology*.

[14] Elkind-Hirsch K., Marrioneaux O., Bhushan M., Vernor D., Bhushan R. (2008). Comparison of single and combined treatment with exenatide and metformin on menstrual cyclicity in overweight women with polycystic ovary syndrome. *Journal of Clinical Endocrinology and Metabolism*.

[15] Tao T., Zhang Y., Zhu Y. C. (2021). Exenatide, metformin, or both for prediabetes in PCOS: a randomized, open-label, parallel-group controlled study. *Journal of Clinical Endocrinology and Metabolism*.

[16] Tang L., Yuan L., Yang G. (2019). Changes in whole metabolites after exenatide treatment in overweight/obese polycystic ovary syndrome patients. *Clinical Endocrinology*.

[17] Zhang Z., Liu Y., Lv J. (2021). Differential lipidomic characteristics of children born to women with polycystic ovary syndrome. *Frontiers in Endocrinology*.

[18] Sun L., Hu W., Liu Q. (2012). Metabonomics reveals plasma metabolic changes and inflammatory marker in polycystic ovary syndrome patients. *Journal of Proteome Research*.

[19] Li S. X., Chu Q. Q., Ma J. (2017). A pilot study of the opposing effects of hyperinsulinemia and hyperandrogenenism on serum lipid profiles and bioactive lipids in women with polycystic ovary syndrome. *Chinese Journal of Endocrinology and Metabolism*.

[20] Ridgway N. (2013). The role of phosphatidylcholine and choline metabolites to cell proliferation and survival. *Critical Reviews in Biochemistry and Molecular Biology*.

[21] Peter C., Waibel M., Keppeler H. (2012). Release of lysophospholipid ’find-me’ signals during apoptosis requires the ATP-binding cassette transporter A1. *Autoimmunity*.

[22] Yao L., Wang Q., Zhang R. (2021). Brown adipose transplantation improves polycystic ovary syndrome-involved Metabolome remodeling. *Frontiers in Endocrinology*.

[23] Atiomo W., Daykin C. A. (2012). Metabolomic biomarkers in women with polycystic ovary syndrome: a pilot study. *Molecular Human Reproduction*.

[24] Szeliga A., Podfigurna A., Bala G., Meczekalski B. (2020). Kisspeptin and neurokinin B analogs use in gynecological endocrinology: where do we stand?. *Journal of Endocrinological Investigation*.

[25] Gorkem U., Togrul C., Arslan E., Sargin Oruc A., Buyukkayaci Duman N. (2018). Is there a role for kisspeptin in pathogenesis of polycystic ovary syndrome?. *Gynecological Endocrinology*.

[26] Silva Figueiredo P., Carla Inada A., Marcelino G. (2017). Fatty acids consumption: the role metabolic aspects involved in obesity and its associated disorders. *Nutrients*.

[27] Rehman K., Haider K., Jabeen K., Akash M. S. H. (2020). Current perspectives of oleic acid: regulation of molecular pathways in mitochondrial and endothelial functioning against insulin resistance and diabetes. *Reviews in Endocrine and Metabolic Disorders*.

[28] Hu X., Weng X., Tian Y. (2019). Effects of omega-3 polyunsaturated fatty acids on steroidogenesis and cellular development in PCOS rats. *Food and Function*.

[29] Ma X., Weng X., Hu X. (2019). Roles of different n-3/n-6 PUFA ratios in ovarian cell development and steroidogenesis in PCOS rats. *Food and Function*.

[30] Di F., Liu J., Li S. (2018). ATF4 contributes to ovulation via regulating COX2/PGE2 expression: a potential role of ATF4 in PCOS. *Frontiers in Endocrinology*.

[31] Nicolas C., Martin O. (2018). Glycoside mimics from glycosylamines: recent progress. *Molecules*.

[32] Jia C., Xu H., Xu Y., Xu Y., Shi Q. (2019). Serum metabolomics analysis of patients with polycystic ovary syndrome by mass spectrometry. *Molecular Reproduction and Development*.

[33] Fiorucci S., Rizzo G., Donini A., Distrutti E., Santucci L. (2007). Targeting farnesoid X receptor for liver and metabolic disorders. *Trends in Molecular Medicine*.

[34] Fiorucci S., Mencarelli A., Palladino G., Cipriani S. (2009). Bile-acid-activated receptors: targeting TGR5 and farnesoid-X-receptor in lipid and glucose disorders. *Trends in Pharmacological Sciences*.

[35] Zhang S., Liu Q., Wang J., Harnish D. C. (2009). Suppression of interleukin-6-induced C-reactive protein expression by FXR agonists. *Biochemical and Biophysical Research Communications*.

